# The impact of mobile game genre on gaming disorder risk in early adolescents: a goal-oriented classification approach

**DOI:** 10.3389/fpsyg.2026.1725287

**Published:** 2026-04-08

**Authors:** KaHee Kim, Sangha Lee, Sowon Hwang, Yunmi Shin

**Affiliations:** 1 Department of Psychiatry, College of Medicine, Ajou University, Suwon, Republic of Korea; 2 Department of Medical Sciences, Graduate School of Ajou University, Suwon, Republic of Korea

**Keywords:** daily gaming time, early adolescence, game genre, gaming disorder, mobile gaming

## Abstract

**Introduction:**

This study aimed to investigate the relationship between mobile game genres and Internet gaming disorder (IGD) in early adolescents using a novel goal-oriented classification system. Additionally, the study examined whether daily gaming time mediates the association between game genre and problematic gaming.

**Methods:**

Data were drawn from Wave 8 of the Kids Cohort for Understanding Internet Addiction Risk Factors in Early Childhood (K-CURE), including 152 participants aged 9 to 12 years. Participants identified their most frequently played mobile game, which was categorized into three groups: physical obstacle, cognitive obstacle, and competitive games. Problematic gaming was assessed using the Internet Gaming Use-Elicited Symptom Screen (IGUESS). Multiple linear regression analyses were performed to evaluate the associations between game genres and IGD, controlling for demographic and parental variables. Mediation analyses were conducted using daily gaming time as a mediator, testing both direct and indirect effects.

**Results:**

Regression analyses indicated that playing physical obstacle games was significantly associated with higher IGUESS scores compared to minimal players (B = 1.709, *p* = 0.039), while cognitive and competitive games did not show significant direct associations. Females scored lower than males (B = −1.462, *p* = 0.014) and older age was a significant predictor (B = 0.092, *p* = 0.005). Mediation analyses revealed that both physical obstacle (indirect *β* = 0.104, *p* = 0.017) and competitive games (indirect *β* = 0.076, *p* = 0.034) had significant indirect effects on IGUESS scores through increased daily gaming time. No direct effects of game genre remained significant after accounting for gaming time, suggesting a pattern consistent with full statistical mediation.

**Discussion:**

These exploratory findings suggest that certain mobile game genres, particularly physical obstacle and competitive games, may be associated with increased risk of problematic gaming in adolescents primarily through their capacity to prolong gaming time rather than through direct effects.

## Introduction

With the revision of the International Classification of Diseases, 11th edition, by the World Health Organization, gaming disorders have been officially recognized as clinical diagnoses and have garnered considerable attention in the field of mental health. Problematic gaming among adolescents has emerged as a significant concern as media use continues to rise globally (Purwaningsih and Nurmala, 2021). Studies have shown that problematic gaming is associated with an increased risk of substance use and higher levels of depression, loneliness, and social anxiety (Archer, 2018; Chen and Zhang, 2023). Adolescents who are vulnerable to impulsivity and have underdeveloped emotional regulation abilities may be particularly susceptible to Internet gaming disorder (IGD) (Chandradasa and Rodrigo, 2017).

In several studies, the severity of problematic gaming in the general public has often been perceived as proportional to the amount of time spent gaming. While gaming time alone does not fully capture patterns of play, types of games, or the subjective experiences of players, it remains an important factor that interacts with other variables in the development of problematic gaming. Accordingly, recent research has increasingly emphasized the need to consider both quantitative indicators such as gaming duration and qualitative aspects such as game genre when examining IGD (Chang and Kim, 2020; Kuss and Griffiths, 2012; Kim et al., 2022). Accordingly, repeated claims have been made focusing on players’ subjective experiences, physiological responses, and levels of immersion in games. As these factors are more strongly affected by game genre or content rather than by gaming time itself, attention has increasingly shifted toward the qualitative aspects of gaming.

Several studies have investigated the association between game genre and IGD based on the qualitative aspects of gaming behavior (Kim et al., 2022; Jo, 2025; Na et al., 2017). Most studies have classified game genres according to player behavior, such as role-playing games (RPG), first-person shooters (FPS), real-time strategy (RTS) games, racing games, arcade games, and shooting games. Many studies have reported that game genres, such as RPG, FPS, and RTS, are associated with a higher risk of problematic gaming.

However, a recurring criticism of these studies is the lack of clarity in the classification of game genres (Clarke and Lee, 2015). Genre classification in gaming research has been criticized for being overly simplistic and failing to capture the complex hybrid nature of modern games (Apperley, 2006). This complexity necessitates more theoretically grounded approaches to genre classification to better capture the relationship between game design features and addictive behaviors. However, the conventional classification methods have several limitations. First, the boundaries between game genres are often ambiguous; for example, a single game may incorporate elements of both the action and role-playing genres. Second, what a player does within a game is subjective; therefore, even when playing the same game, the player’s experience may differ, making it difficult to regard genre classification as objective. Therefore, there is an increasing need for a more refined and precise classification of game genres.

To address these problems, this study employed a novel approach to classify game genres instead of relying on conventional methods. According to Lee and Kwon (2008), games can be broadly categorized based on whether they aim for individual goal attainment or structured competition. Participants were asked to identify their most frequently played mobile game, which was then categorized by trained researchers using predetermined criteria based on game mechanics and objectives. This approach proposes a more objective system that does not depend on the player’s subjective experience. In this study, mobile games were classified into three categories–physical obstacle games, cognitive obstacle games, and competitive games–and the associations between these categories and IGD were analyzed. By classifying game genres according to a consistent set of criteria rather than simply listing genres as in conventional classification methods, this study aimed to examine more clearly how game characteristics are associated with problematic gaming. Additionally, the study examined whether daily gaming time mediates the relationship between game genre and IGD.

## Methods

### Study design and participants

This study utilized data from Wave 8 of the Kids Cohort for Understanding Internet Addiction Risk Factors in Early Childhood (K-CURE). The K-CURE represents the first prospective study in South Korea to investigate the long-term impacts of early media exposure on child development. Since its inception in 2015, it continuously tracked the developmental trajectories of children aged 2–5 years annually through parent-reported assessments. Notably, self-reported assessments were introduced for the first time in 2022, as the participants had reached the 4th grade of elementary school at approximately 10 years old.

Data were collected using a web-based electronic Case Report Form system. Children completed the online survey individually, either at home or at school, taking approximately 30–40 min per assessment.

A total of 164 children participated in this survey. After excluding 12 participants with missing data on key variables (parental ages, *n* = 5; observer-reported parental internet addiction scores, *n* = 7), 152 participants were included in the final analysis. Participants who did not report a specific most-frequently-played mobile game were classified as “minimal players” and served as the reference category in all regression and mediation analyses. The participants’ ages at the time of the survey ranged from 9 years, 11 months to 12 years, 7 months (mean = 11.22 years, SD = 0.72). The sample consisted of 92 boys (60.5%) and 60 girls (39.5%). The survey encompassed various aspects including smart device usage patterns, gaming and social media behaviors, mental and emotional health, and sleep habits. To complement subjective self-reported data with objective behavioral metrics, participants were also requested to upload screen time captures from their primary devices.

### Assessment tools

This study employed several validated self-reported and observer-reported instruments to assess digital addiction tendencies among adolescents and their parents.

The Adolescent Internet Addiction Self-Diagnosis Scale: Brief Form (KS-II) was used to evaluate adolescents’ risk of Internet addiction. Developed as part of the “Advanced Research on Internet Addiction Diagnostic Scale” by Shin and Kim (2011a), the KS-II consists of 15 items based on the original K scale (Kim et al., 2002), as well as theoretical frameworks proposed by Young (1996) and Greenfield (1999). The scale demonstrated high internal consistency (Cronbach’s *α* = 0.814), and its criterion and factor validity were supported through AMOS-based analysis.

To measure adolescents’ tendencies toward problematic smartphone use, the Smartphone Addiction Proneness Scale for Youth: Self-Report (S-scale) was administered. This 15-item scale was developed by Shin and Kim (2011b) in a national project on smartphone addiction. It showed strong internal consistency (Cronbach’s *α* = 0.880) and acceptable levels of both criterion and structural validity.

Problematic Internet gaming behavior was assessed using the Internet Gaming Use-Elicited Symptom Screen (IGUESS), a 9-item screening instrument developed by Cho and Lee (2013). Based on the Diagnostic and Statistical Manual of Mental Disorders criteria, the IGUESS is suitable for children and adults and exhibited excellent reliability (Cronbach’s *α* = 0.940). At a cutoff score of 8, the IGUESS demonstrated a sensitivity of 91% and a specificity of 87%. At a cutoff score of 10, the sensitivity was 79% and the specificity was 87%.

The Short-Form Internet Addiction Proneness Scale for Adults: Self-Report was used for parents. This 15-item tool is based on the original K scale and demonstrated robust internal reliability (Cronbach’s *α* = 0.870).

Finally, to assess the digital behavior of the adolescent’s other parent as observed by their partner, the Internet Addiction Proneness Scale for Adults: Observer-Report was administered. This 15-item scale incorporates elements from Young (1996); Greenfield (1999), and the K-scale. It showed moderate internal consistency (Cronbach’s *α* = 0.711), with structural validity confirmed through factor analysis.

### Game genre classification

**Figure 1:**
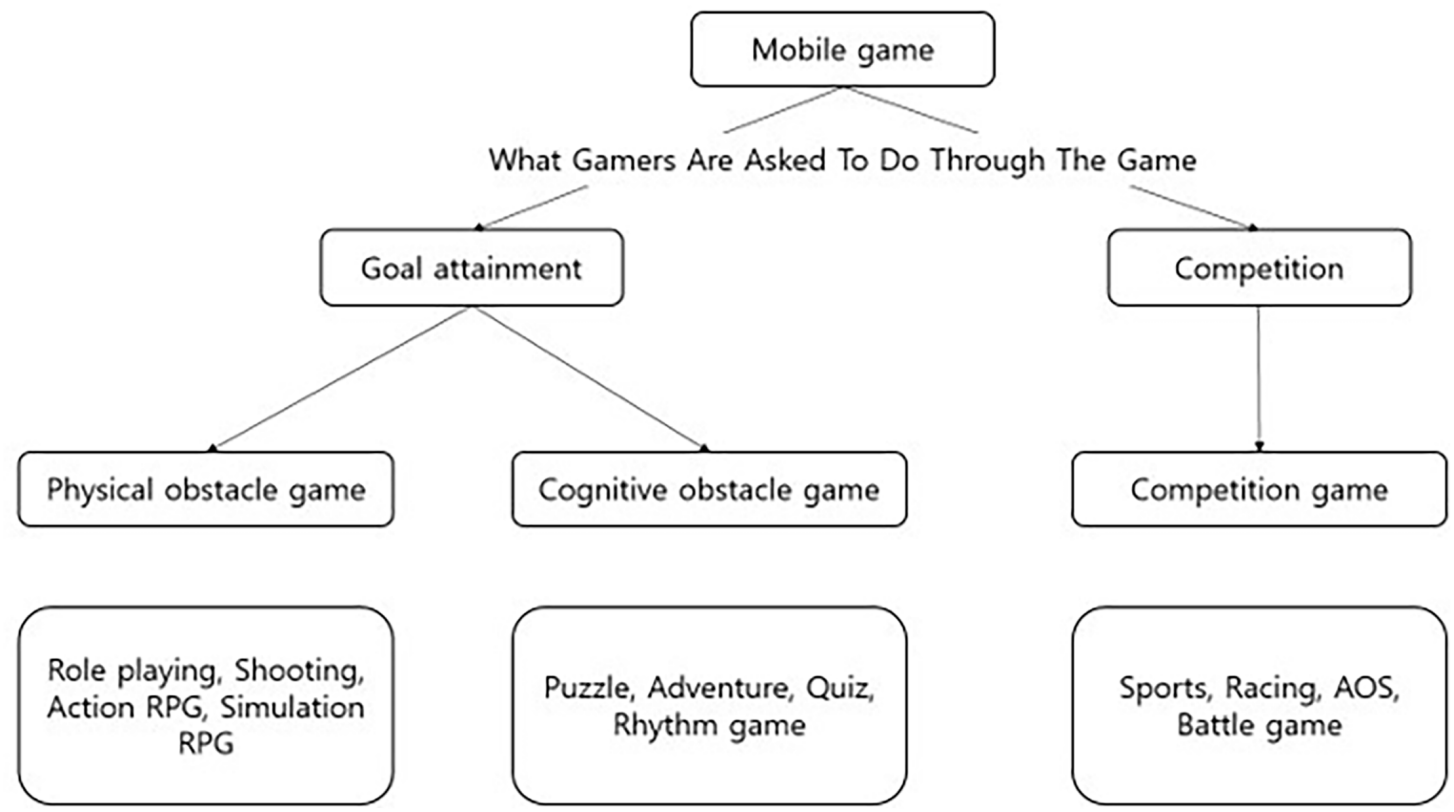
Classification of game genres. Mobile games were categorized into three groups according to game mechanics and objectives: physical obstacle games (requiring motor coordination and reflexive action, e.g., role-playing, shooting, action RPG), cognitive obstacle games (requiring problem-solving and pattern recognition, e.g., puzzle, adventure, quiz, rhythm games), and competitive games (involving structured competition, e.g., sports, racing, AOS, battle games), based on the framework by Lee and Kwon (2008).

Participants were asked to identify their most frequently played mobile game from a standardized list of 21 genre options. Based on the goal-oriented classification framework by Lee and Kwon (2008), reported genres were reclassified into three categories: physical obstacle games (requiring motor coordination and reflexive action, e.g., RPG, shooting, action), cognitive obstacle games (requiring problem-solving and pattern recognition, e.g., puzzle, adventure, rhythm), and competitive games (involving structured competition against opponents governed by shared rules, e.g., sports, racing, AOS) (Figure 1). Classification was performed by trained researchers using predefined criteria based on each game’s primary gameplay mechanic; when a game contained hybrid elements, the primary mechanic determined its category. A complete list of sub-genres and their assigned categories is provided in Supplementary Table S1.

### Statistical analyses

All statistical analyses were conducted using Jamovi software (Version 2.6.26), which is based on the R statistical computing environment (R Core Team, 2024). The data analysis proceeded as follows: First, descriptive statistics, including means and standard deviations, were computed to understand the overall characteristics and distribution of the key variables. Subsequently, to identify significant differences between the various groups, a one-way analysis of variance was used. Specifically, Welch’s correction was applied when the assumption of homogeneity of variances was violated. When statistically significant differences were found, appropriate post-hoc comparisons were conducted using the Games–Howell test, which is suitable for unequal variances and sample sizes.

Multiple linear regression analyses were performed to identify the key factors predicting IGUESS scores among adolescents. In these analyses, the independent variables included demographic factors (age, sex, parental age, and monthly household income), media use patterns (mobile game initiation age and game genre group), and parental internet use characteristics (log-transformed scores of adult internet addiction and observer-reported parental internet addiction).

To examine whether daily gaming time mediates the association between game genre and IGD, mediation analyses were conducted using the jAMM (Jamovi Advanced Mediation Models) module in Jamovi (Version 2.6.26). Daily gaming time was calculated as a weighted average: [(weekday hours × 5) + (weekend hours × 2)]/7, incorporating both child-appropriate and 12 + rated games measured on a 7-point scale. The mediation model included game genre groups as predictors, daily gaming time as the mediator, and IGUESS scores as the outcome, controlling for all demographic and parental variables included in the regression analyses. We evaluated path a (genre → time), path b (time → IGUESS), path c′ (direct effect: genre → IGUESS), and the indirect effect (a × b). Confidence intervals were computed using the Delta method, with *α* = 0.05. We note that while bootstrapping is increasingly recommended for mediation analyses, the Delta method was employed as the default procedure in jAMM and was considered appropriate given the moderate sample size (*N* = 152). As the current analysis is cross-sectional, the mediation model does not establish temporal ordering or causal direction.

### Ethics statement

This study received approval from the Institutional Review Board (IRB) at the Ajou University School of Medicine, Suwon City, South Korea (IRB No. AJIRB-SBR-SUR-22-285). Written informed consent was obtained from all participants’ legal guardians prior to enrollment, and written assent was obtained from the child participants themselves. Both guardians and children were informed of the study’s purpose, procedures, expected duration, and their right to withdraw from the study at any time without penalty. All data were collected via a secure, password-protected electronic Case Report Form system. Personal identifiers were removed prior to analysis, and all data were stored on secure servers accessible only to authorized research personnel to ensure confidentiality.

## Results

This study analyzed the association between game genre and problematic gaming among early adolescents. After excluding 12 participants with missing data on key variables, the final analytic sample consisted of 152 participants, of whom 92 (60.5%) were male and 60 (39.5%) were female. Participants were classified into four groups based on their most frequently played mobile game: 59 (38.8%) played physical obstacle games, 41 (27.0%) played cognitive obstacle games, 30 (19.7%) played competitive games, and 22 (14.5%) were minimal players (i.e., participants who did not report a specific most-frequently-played mobile game). The mean participant age was 11.22 years (SD = 0.72), with no significant age differences observed across groups. Participants reported beginning mobile game use at an average age of 8.77 years (SD = 1.50). The mean ages of fathers and mothers were 45.33 years (SD = 4.79) and 42.79 years (SD = 3.59), respectively. A chi-square test revealed a significant association between sex and game genre group, *χ*^2^(3) = 17.16, *p* = 0.001. Male participants were substantially more likely to play competitive games than female participants (27.1% vs. 5.9%), whereas female participants were more likely to be classified as minimal players (25.0% vs. 8.3%). The proportions of physical obstacle and cognitive obstacle game players were similar across sexes. Overall, the sample was evenly distributed across various demographic parameters (Table 1).

**Table 1:** Demographic characteristics of participants by game genre group.

Demographics	Minimal player (*n* = 22)	Physical obstacle (*n* = 59)	Cognitive obstacle (*n* = 41)	Competitive game (*n* = 30)	Total (*N* = 152)
Sex					
Male	8 (36.4%)	34 (57.6%)	24 (58.5%)	26 (86.7%)	92 (60.5%)
Female	14 (63.6%)	25 (42.4%)	17 (41.5%)	4 (13.3%)	60 (39.5%)
Age (years)	11.16 ± 0.71	11.39 ± 0.74	11.10 ± 0.69	11.09 ± 0.71	11.22 ± 0.72
Parent’s age (years)					
Father	46.23 ± 4.60	45.71 ± 5.56	44.78 ± 4.02	44.67 ± 4.26	45.33 ± 4.79
Mother	43.59 ± 3.05	43.73 ± 3.82	42.05 ± 3.32	41.37 ± 3.27	42.79 ± 3.59
Age at first mobile game use (years)	8.91 ± 1.63	8.80 ± 1.41	8.76 ± 1.66	8.63 ± 1.40	8.77 ± 1.50
Monthly house hold income					
Level 1	0 (0.0%)	2 (3.4%)	0(0.0%)	0(0.0%)	2 (1.3%)
Level 2	3 (13.6%)	7 (11.9%)	4 (9.8%)	5 (16.7%)	19 (12.5%)
Level 3	7 (31.8%)	21 (35.6%)	21 (51.2%)	13(43.3%)	62 (40.8%)
Level 4	12 (54.6%)	29 (49.1%)	16 (39.0%)	12(40.0%)	69 (45.4%)
Daily game play time (hours)	0.22 ± 0.47	2.09 ± 1.84	1.49 ± 2.01	1.95 ± 2.04	1.63 ± 1.90

Values are presented as mean ± SD or *n* (%).

Monthly household income levels were classified as follows: Level 1 = <2,000,000 KRW (≈ USD 1,550); Level 2 = 2,000,000–3,999,999 KRW (≈ USD 1,550–3,100); Level 3 = 4,000,000–5,999,999 KRW (≈ USD 3,100–4,650); Level 4 = ≥6,000,000 KRW (≈ USD 4,650).

Currency values were converted using the average exchange rate in 2022 (1 USD = 1,291 KRW).

Welch’s ANOVA indicated a significant difference in IGUESS scores across game genre groups, *F*(3, 78.5) = 4.99, *p* = 0.003. Games–Howell post-hoc tests revealed that the physical obstacle game group had significantly higher IGUESS scores than minimal players (*p* = 0.002), while the cognitive obstacle and competitive game groups did not differ significantly from minimal players (Table 2).

**Table 2:** Mean differences in IGUESS scores and daily gaming time by game genre.

Game genre group	*N*	Mean	SD	Post-hoc (vs. non-player)	*p*
IGUESS score					
Non-player	25	1.16	1.89	—	—
Physical obstacle	65	3.40	3.85	Significant	**0.002**
Cognitive obstacle	44	2.36	3.58	N.S.	0.270
Competitive game	30	2.90	3.49	N.S.	0.101
Daily gaming time					
Non-player	25	0.22	0.55	—	—
Physical obstacle	65	1.71	1.68	Significant	**<0.001**
Cognitive obstacle	44	1.13	1.76	Significant	**0.003**
Competitive game	30	1.57	1.61	Significant	**<0.001**

Post-hoc: Games–Howell test. Bold indicates statistically significant difference (*p* < 0.05). IGUESS: Welch’s ANOVA F(3, 78.5) = 4.99, *p* = 0.003. IGUESS, internet gaming use-elicited symptom screen total score. Daily gaming time: Welch’s ANOVA F(3, 78.7) = 17.55, *p* < 0.001. Daily gaming time = weighted average of daily gaming hours [(weekday hours × 5 + weekend hours × 2)/7].

Multiple linear regression analysis was conducted to predict IGUESS scores (Table 3). The overall model accounted for approximately 12.5% of the variance in IGUESS scores (Adjusted *R*^2^ = 0.125). Significant predictors included the game group, participant sex, and age in months. Specifically, playing physical obstacle games was associated with higher IGUESS scores compared to minimal players (B = 1.709, *p* = 0.039). Other game genres (cognitive obstacle games and competitive games) did not show statistically significant differences compared to minimal players. Regarding demographic variables, females scored significantly lower than males on the IGUESS (B = −1.462, *p* = 0.014), indicating that males exhibited higher levels of problematic gaming. Additionally, age was a significant positive predictor (B = 0.092, *p* = 0.005), indicating that older participants tended to have higher IGUESS scores.

**Table 3:** Factors associated with internet gaming addiction.

			95% Confidence interval			
Predictor	*B*	*SE*	Lower	Upper	*t*	*p*
Intercept^a^	−22.164	6.968	−35.939	−8.389	−3.181	**0.002**
Group						
2–1	1.709	0.821	0.087	3.332	2.083	**0.039**
3–1	1.169	0.876	−0.562	2.900	1.335	0.184
4–1	1.271	0.962	−0.631	3.173	1.321	0.189
Sex						
M–F	−1.462	0.588	−2.625	−0.300	−2.486	**0.014**
Age (years)	1.106	0.388	0.341	1.872	2.857	**0.005**
Father age (years)	0.115	0.074	−0.031	0.261	1.554	0.122
Mother age (years)	0.001	0.101	−0.198	0.199	0.008	0.994
Monthly household income	−0.065	0.367	−0.791	0.662	−0.175	0.861
Log-transformed adult internet addiction	1.414	1.114	−0.789	3.616	1.269	0.207
Observer-reported parental internet addiction	0.539	1.292	−2.015	3.094	0.417	0.677
Age at first mobile game use	0.074	0.184	−0.291	0.438	0.400	0.690
Adjusted *R*^2^	0.125					

1: Minimal players (reference group); 2: Physical obstacle game group; 3: Cognitive obstacle game group; 4: Competitive game group. Sex was coded as 1 = Male (reference) and 2 = Female. The contrast “Female–Male” indicates that the coefficient represents the difference in IGUESS scores for females relative to males. B = unstandardized regression coefficient. Bold indicates statistically significant difference (*p* < 0.05).

a Unstandardized coefficients.

**Figure 2:**
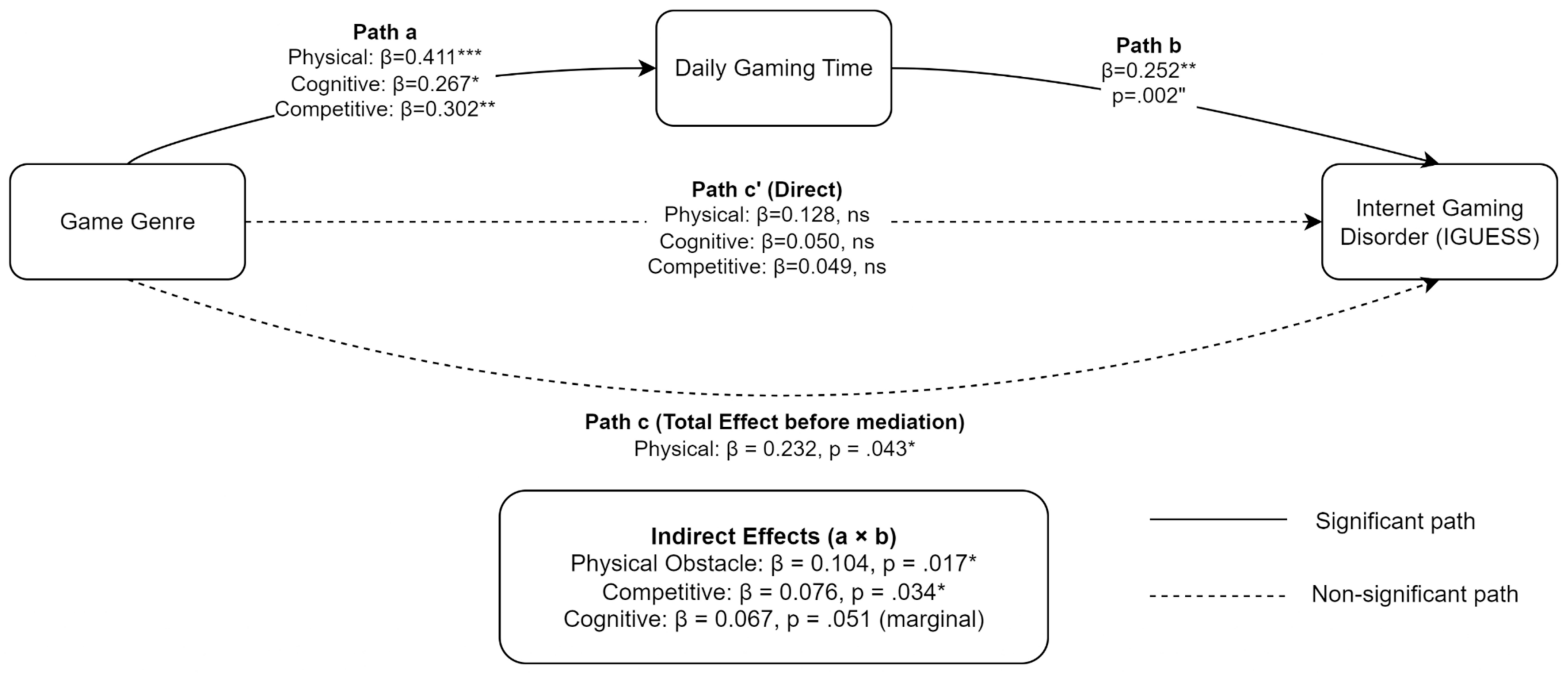
Mediation model of daily gaming time between game genre and internet gaming disorder (IGD). Path coefficients (standardized *β*) are presented for associations between game genres, daily gaming time, and IGUESS scores. All game genre groups are compared to minimal players (reference group). Solid lines indicate statistically significant paths (*p* < 0.05), and dashed lines indicate non-significant paths. Confidence intervals were computed using the Delta method. *p < 0.05. **p < 0.01, ***p < 0.001. IGUESS, internet gaming use-elicited symptom screen.

To further explore the associations underlying the relationship between game genre and problematic gaming, mediation analysis was conducted with daily gaming time as a mediator (Table 4, Figure 2). The analysis revealed significant indirect effects of game genre on IGUESS scores through daily gaming time. Specifically, physical obstacle games exhibited a significant indirect effect (indirect *β* = 0.104, *p* = 0.017), as did competitive games (indirect *β* = 0.076, *p* = 0.034). The indirect effect for cognitive obstacle games approached but did not reach conventional statistical significance (*β* = 0.067, *p* = 0.051).

**Table 4:** Mediation analysis: gaming time as mediator of genre effects on gaming addiction.

Effect type	Path	B	SE	95% CI lower	95% CI upper	*β*	p
Indirect effects							
	Physical obstacle → time → IGUESS	0.730	0.305	0.131	1.328	0.104	**0.017**
	Cognitive obstacle → time → IGUESS	0.520	0.266	−0.002	1.041	0.067	0.051
	Competitive → time → IGUESS	0.657	0.311	0.048	1.266	0.076	**0.034**
Path coefficients							
Path a	Physical obstacle → gaming time	1.597	0.432	0.75	2.443	0.411	**<0.001**
	Cognitive obstacle → gaming time	1.137	0.455	0.245	2.029	0.267	**0.013**
	Competitive → gaming time	1.437	0.502	0.454	2.420	0.302	**0.004**
Path b	Gaming time → IGUESS	0.457	0.146	0.171	0.743	0.252	**0.002**
Direct effects							
Path c′	Physical obstacle → IGUESS	0.900	0.811	−0.69	2.490	0.128	0.267
	Cognitive obstacle → IGUESS	0.382	0.835	−1.255	2.019	0.05	0.647
	Competitive → IGUESS	0.419	0.926	−1.396	2.233	0.049	0.651
Total effects							
Path c	Physical obstacle → IGUESS	1.630	0.805	0.053	3.207	0.232	**0.043**
	Cognitive obstacle → IGUESS	0.902	0.848	−0.759	2.563	0.117	0.287
	Competitive → IGUESS	1.076	0.934	−0.754	2.905	0.125	0.249
Control variables							
	Age → IGUESS (direct)	0.080	0.031	0.020	0.140	0.203	**0.009**
	Sex (M vs. F) → IGUESS (direct)	−1.002	0.556	−2.092	0.088	−0.143	0.072

Gaming time = weighted average of daily gaming hours [(weekday hours × 5 + weekend hours × 2)/7] including both child-appropriate and 12 + rated games.

Indirect, direct, and total effects of each game genre on IGUESS scores are reported, with daily gaming time as a mediator. Standardized path coefficients (*β*), standard errors (SE), confidence intervals (CI), and *p* values are presented. IGUESS, internet gaming use-elicited symptom screen.

Bold indicates statistically significant difference (*p* < 0.05).

All game genre groups compared to minimal players (reference group).

IGUESS, internet gaming use-elicited symptom screen. *β*, completely standardized effect size.

Confidence intervals computed using standard (Delta method).

B = unstandardized regression coefficient.

Path analyses demonstrated that all game genres were associated with increased daily gaming time compared to minimal players, with physical obstacle games showing the strongest association (*β* = 0.411, *p* < 0.001), followed by competitive (*β* = 0.302, *p* = 0.004) and cognitive obstacle games (*β* = 0.267, *p* = 0.013). Daily gaming time significantly predicted IGUESS scores (*β* = 0.252, *p* = 0.002).

When gaming time was included in the model, direct effects of all genres became non-significant (all *p* > 0.05), yielding a pattern consistent with full statistical mediation in the cross-sectional data.

## Discussion

This study analyzed the association between game genres and problematic gaming among early adolescents aged 9 to 12 years. The IGUESS scores were higher in the physical obstacle games group than in the other groups. Multiple linear regression analysis revealed that physical obstacle games were significantly associated with higher IGUESS scores. In addition to game genre, sex and age were also found to be significant predictors of IGUESS scores. However, parental internet addiction—assessed through both self-report and partner-observation measures—did not significantly predict adolescents’ IGUESS scores in the current model.

Previous studies have reported that genres such as RPGs, FPSs, and RTS games are associated with a higher risk of problematic gaming (Kim et al., 2022). While our initial findings appeared to align with this pattern—physical obstacle games (which include RPGs and RTS) showed elevated IGUESS scores—our mediation analysis revealed a different underlying pattern. Rather than being directly associated with higher IGD risk through genre-specific features alone, these games appear to be associated with increased problematic gaming primarily through their capacity to extend play duration. This shifts understanding from “which genres are associated with problematic gaming” to ‘how games may relate to problematic gaming through prolonged engagement’.

The mediation analysis further clarified the associations between game genres and problematic gaming. Physical obstacle and competitive games were not directly associated with IGUESS scores after accounting for daily gaming time (path c′: both *p* > 0.05), but both were significantly associated with increased gaming duration (path a: *p* < 0.01), which in turn was associated with elevated IGUESS scores (path b: *β* = 0.252, *p* = 0.002). Cognitive obstacle games showed a similar pattern, though the indirect effect was marginal (*p* = 0.051).

From a theoretical perspective, this pattern is consistent with a model in which the risk of IGD is best understood through the intervening role of gaming duration rather than through simple associations between genre and problematic gaming. Different game genres appear to vary primarily in their capacity to initiate and sustain gaming behavior. However, it should be noted that these cross-sectional findings cannot establish causal directionality, and longitudinal analyses from future K-CURE waves will be needed to test temporal ordering.

These associations may be related to specific game design features, including the level of game immersion, in-game competition, reward systems, and mechanisms of social interaction, all of which may prolong the amount of time players spend in the game and thereby be associated with increased risk of problematic gaming. In both physical obstacle and competitive games, vivid imagery and sound are often used to foster deeper immersion, while the formation of in-game networks encourages players to remain engaged for longer periods. Features that allow players to immediately engage in team play upon logging in, along with the excitement and sense of achievement gained during play, may further contribute to prolonged gaming sessions. Moreover, these games stimulate dopamine release and enhance sustained interest by offering a carefully calibrated balance of continuous and variable reinforcement. Modern game design also increasingly incorporates monetization features, such as loot boxes and near-miss mechanics, which further reinforce prolonged engagement and may enhance addictive potential (King and Delfabbro, 2018; Larche et al., 2017). These design elements, commonly found in both physical obstacle and competitive games, may therefore help explain why these genres are associated with elevated IGUESS scores through their capacity to extend gaming duration.

The findings showed a significant association between age and IGUESS scores, which may be due to the tendency of older adolescents to engage more frequently in physical obstacle games than in cognitive obstacle games. Sex also emerged as a significant factor; a chi-square analysis confirmed that game genre preference differed significantly by sex (*χ*^2^(3) = 17.16, *p* = 0.001), with male participants being substantially more likely to play competitive games (27.1% vs. 5.9%) and less likely to be minimal players (8.3% vs. 25.0%) compared to female participants. This pattern of greater engagement in active gaming among males is consistent with previous studies reporting that males are generally more vulnerable to IGD than females (Ko et al., 2005; Nisha et al., 2024).

This study has several limitations. First, the analysis was based on a relatively small sample size of 152 participants (164 before exclusions). Although the 12 excluded participants had complete data on most study variables, a consistent analytic sample (*N* = 152) was used across all regression and mediation analyses to ensure comparability. Sensitivity checks confirmed that descriptive and ANOVA results using the full sample (*N* = 164) were substantively identical to those obtained with the analytic sample. The modest adjusted *R*^2^ of 0.125 indicates that approximately 87.5% of the variance in IGUESS scores remains unexplained by the current model. This likely reflects the multifactorial nature of problematic gaming, and future studies should consider additional predictors such as individual psychological factors (e.g., impulsivity, self-control, emotion regulation), social and peer influences on gaming behavior, exposure to specific game monetization features, and family factors beyond parental internet addiction (e.g., parenting style, parent–child relationship quality). Second, although the mediation analysis revealed a pattern consistent with full statistical mediation, the cross-sectional nature of the current analysis precludes conclusions about causal direction or temporal ordering. Longitudinal data from future K-CURE waves will be necessary to test whether the observed associations reflect causal pathways. Third, the IGUESS is a screening tool, not a diagnostic instrument for gaming disorders; thus, classifying individuals as having IGD based solely on their scores may not be accurate. Fourth, confidence intervals for indirect effects were computed using the Delta method rather than bootstrapping; future analyses should incorporate bootstrap resampling to further validate the stability of the indirect effect estimates. Additionally, the novel genre classification system used in this study, while theoretically grounded in the framework of Lee and Kwon (2008), has not been empirically validated against established gaming behavior measures, and formal inter-rater reliability was not calculated. Although all classifications were performed by trained researchers using predefined criteria and discrepancies were resolved through group discussion, future studies should incorporate independent double-coding with inter-rater reliability reporting to strengthen methodological rigor. The current findings should therefore be considered exploratory in nature. Furthermore, the non-significance of direct effects after accounting for gaming time does not necessarily indicate the absence of genre-specific psychological mechanisms, which may operate through pathways not captured in the current model.

## Conclusion

In conclusion, this study classified game genres using a clear and consistent goal-oriented framework and identified daily gaming time as a potential intervening variable through which game genre is associated with problematic gaming risk. While physical obstacle and competitive games showed significant indirect associations with IGUESS scores through gaming duration, direct genre effects were not significant when gaming time was accounted for. From a clinical standpoint, these exploratory findings emphasize the potential importance of monitoring and managing gaming duration among adolescents who engage in physical obstacle and competitive games. Rather than focusing solely on the categorical restriction of particular game types, interventions targeting the regulation of play time may be relevant in mitigating the risk of problematic gaming. Parental supervision and psychoeducational programs promoting time management could serve as preventive strategies. It should be noted that not all adolescents who play high-risk games develop IGD; factors such as self-control, individual personality traits, and parental supervision have been reported as important protective variables (Ropovik et al., 2022). Therefore, further research is needed to examine the relationships between these potential protective factors and problematic gaming, ideally using longitudinal designs that can establish temporal ordering.

## Data Availability

The dataset used in this study is part of the Kids Cohort for Understanding Internet Addiction Risk Factors in Early Childhood (K-CURE), managed by the National Center for Mental Health. Due to ethical restrictions and data protection policies, the dataset cannot be publicly shared or distributed by the authors. Requests to access the datasets should be directed to YS, ymshin@ajou.ac.kr.
