# Supplementary material for: The impact of mobile game genre on gaming disorder risk in early adolescents: a goal-oriented classification approach
Source: Front Psychol. 2026 Apr 8;17:1725287. doi: 10.3389/fpsyg.2026.1725287 (PMC13099844; doi:10.3389/fpsyg.2026.1725287)
Supplement: Supplementary file 1 [file Table_1.DOCX]

**Supplementary Table S1.** Classification of most frequently played mobile games by genre category.

Participants reported their most frequently played mobile game genre from a standardized list of 21 options. These sub-genres were then reclassified into three goal-oriented categories based on the framework by Lee and Kwon (2008): *physical obstacle games* (requiring motor coordination and reflexive action to overcome physical challenges), *cognitive obstacle games* (requiring problem-solving, pattern recognition, or perceptual skills), and *competitive games* (involving structured competition against other players or AI opponents governed by shared rules). Participants who reported no specific game were classified as minimal players.

| **Genre Category** | **Original Survey Sub-genre** | **Representative Game Titles** | **n** |
| --- | --- | --- | --- |
| Physical Obstacle | Shooting (FPS/TPS) | PUBG Mobile, Brawl Stars, etc. | 27 |
| Physical Obstacle | RPG (MORPG/MMORPG) | Lineage M, Cookie Run: Kingdom, Black Desert Mobile, etc. | 21 |
| Physical Obstacle | Action (Running/Side-scrolling) | Traffic Run, Talking Tom Gold Run, Cookie Run, etc. | 9 |
| Physical Obstacle | Real-Time Strategy (RTS) | Clash Royale, Rise of Kingdoms, Random Dice, etc. | 5 |
| Physical Obstacle | Action RPG (ARPG) | Blade & Soul Revolution, Assassin’s Creed, Honkai Impact 3, etc. | 1 |
| Physical Obstacle | Simulation RPG (SRPG) | Clash of Three Kingdoms, Mafia City, etc. | 1 |
| Cognitive Obstacle | Adventure | Light After, Pokémon GO, etc. | 30 |
| Cognitive Obstacle | Rhythm | Club Audition, Love Beat, Tapsonic, etc. | 6 |
| Cognitive Obstacle | Puzzle | Anipang, Candy Crush Saga, Friends Popcorn, Sudoku, etc. | 5 |
| Cognitive Obstacle | Quiz | OX Quiz Survival 100, Koongya Draw Party, Quiz Rumble Connect, etc. | 1 |
| Competitive | Racing | KartRider Rush+, Asphalt, Friends Racing, etc. | 15 |
| Competitive | Sports | PES (Winning Eleven), Com2uS Pro Baseball, FIFA Online M, etc. | 9 |
| Competitive | AOS (MOBA) | League of Legends: Wild Rift, Mobile Legends: Bang Bang, etc. | 4 |
| Competitive | Fighting | King of Fighters, Shadow Fighter 3, Street Fighter, etc. | 2 |
| Minimal Player | — | No specific game reported | 21 |

***Note.*** *n = number of participants reporting each sub-genre as their most frequently played mobile game. Sample sizes are based on the full sample (N = 157) prior to exclusion for missing data. Representative game titles are drawn from the standardized survey options provided to participants. Sub-genres not selected by any participant (e.g., Simulation, Board, Casino, Card, Education, Social Network Games) are not listed. AOS = Aeon of Strife; MOBA = Multiplayer Online Battle Arena; RPG = Role-Playing Game; RTS = Real-Time Strategy; FPS = First-Person Shooter; TPS = Third-Person Shooter.*
